# Predicting enhancer-promoter interactions using a stacking-based ensemble strategy

**DOI:** 10.1093/bioinformatics/btag359

**Published:** 2026-06-04

**Authors:** Zhichao Xiao, Haibo Ji, Quan Zou, Yijie Ding, Liang Yu

**Affiliations:** School of Computer Science and Technology, Xidian University, Xi'an 710126, China; School of Computer Science and Technology, Xidian University, Xi'an 710126, China; Yangtze Delta Region Institute (Quzhou), University of Electronic Science and Technology of China, Quzhou 324000, China; Yangtze Delta Region Institute (Quzhou), University of Electronic Science and Technology of China, Quzhou 324000, China; School of Computer Science and Technology, Xidian University, Xi'an 710126, China

## Abstract

**Motivation:**

Enhancer–promoter interactions (EPIs) are essential for gene regulation and disease progression. Recent studies have shown that distal enhancers can regulate target genes through interactions with nearby promoters, providing important insights into transcriptional regulation mechanisms. Although high-throughput experimental techniques have enabled large-scale identification of EPIs, these methods are often costly and time-consuming. In addition, existing computational approaches still face challenges in effectively integrating heterogeneous feature representations from different cell lines.

**Results:**

We propose a stacked ensemble framework for EPI prediction that integrates feature representations from diverse cell line datasets using multiple machine learning algorithms. The extracted complementary patterns are further combined by an XGBoost classifier to improve robustness against overfitting. Experiments on six independent datasets show that the proposed method achieves superior accuracy and generalization compared with existing EPI prediction models, with an average AUROC of 0.909 while maintaining computational efficiency.

**Availability:**

The source code and its archived release are available at GitHub and Zenodo. The Zenodo archive provides a versioned snapshot of the repository: https://zenodo.org/records/19952998

## 1 Introduction

Enhancer-promoter interactions (EPIs) represent two pivotal regulatory elements governing gene expression in mammalian genomes, with profound implications for human biology. The precise coordination between these elements is critical for maintaining transcriptional fidelity, cellular homeostasis, and developmental trajectories. Furthermore, aberrant spatial associations between enhancers and promoters have been implicated in dysregulated gene expression patterns associated with disease pathogenesis (Schoenfelder and Fraser 2019, Wei *et al.* 2021, Cao *et al*. 2025). Despite the biological significance of EPIs, their genome-wide mechanistic underpinnings remain poorly understood due to the inherent complexity of chromatin interaction networks (Ni *et al*. 2022). The advent of high-throughput genomic techniques, such as Hi-C (Lieberman-Aiden *et al*. 2009) and ChIA-PET (Zhang *et al*. 2012), has revolutionized our ability to dissect the three-dimensional architecture of chromatin interactions, revealing that enhancers frequently bypass proximal promoters to engage in long-range looping with distal targets separated by megabase-scale distances (Field and Adelman 2020). However, the sequence-level determinants governing these spatial associations remain largely enigmatic, necessitating the development of robust computational frameworks to systematically interrogate EPIs (Wei *et al.* 2021).

Recent advancements in computational biology have spurred the emergence of numerous algorithms designed to predict EPIs at scale. Given the voluminous nature of genomic data, most state-of-the-art methods now rely on deep learning paradigms (Zulfiqar *et al*. 2023, Joshi and Singh 2024, Mahapatra *et al*. 2024). Early computational approaches typically integrated multi-omics features, such as DNase I hypersensitivity sites, histone modification profiles, and transcription factor binding data, into supervised learning models. For instance, Whalen *et al.* developed TargetFinder (Whalen *et al*. 2016), which employs a random forest (RF) (Breiman 2001, Ru *et al*. 2019) and support vector machine (SVM) (Hearst *et al*. 1998, Meher *et al*. 2024, Wang *et al*. 2024, Yan *et al*. 2024) classifier trained on integrated genomic datasets to predict functional interactions. Similarly, Cao *et al.* developed ChINN (Cao *et al*. 2021), which leverages convolutional neural networks (CNNs) to enable genome-wide EPI prediction.

In recent years, there has been a paradigm shift toward sequence-based modeling strategies (Li *et al*. 2024, Ma *et al*. 2024, Ren *et al*. 2024). This trend is driven by two key advantages: (1) genomic sequence data requires minimal preprocessing compared to heterogeneous omics datasets, and (2) innovations in natural language processing (NLP) have provided sophisticated tools for sequence analysis (Wei *et al.* 2021). Yang *et al.* pioneered PEP-Word (Yang *et al*. 2017), which employs word embedding techniques to extract sequence-derived features and trains gradient-boosted tree ensembles for EPI prediction, demonstrating that sequence alone can reliably predict interaction landscapes. Concurrently, Mao et al.'s EPIANN (Mao *et al*. 2017) introduced attention mechanisms coupled with positional encoding to enhance predictive accuracy. Hybrid architectures combining CNNs with recurrent neural networks (RNNs) have further advanced this field; examples include Singh et al.'s SPEID (Singh *et al*. 2019), which integrates CNNs with long short-term memory (LSTM) networks, and Liu et al.'s EPIVAN (Hong *et al*. 2020), which combines CNNs with gated recurrent units (GRUs) and employs pre-training strategies.

Despite these advancements, current deep learning models still face several challenges in EPI prediction (Liu *et al*. 2024). In particular, many approaches, including deep learning-based models, rely on a single modeling paradigm and may not fully exploit the complementary information embedded in heterogeneous feature representations. In addition, although deep learning methods can learn complex patterns, their implicit feature learning process may not always effectively incorporate domain-specific knowledge. These limitations can restrict the model’s ability to capture diverse regulatory patterns and affect its robustness and generalizability across different cell lines (Manavalan *et al*. 2019, Li *et al*. 2021, Liang *et al*. 2024, Yin *et al*. 2024, Wang *et al*. 2025). Effectively integrating multi-source features to improve predictive performance remains an important challenge in this field.

To address the aforementioned challenges, we propose a novel stacking ensemble framework to systematically integrate and reconstruct feature representations across diverse cell-line datasets. This approach employs multiple machine learning algorithms as base classifiers, each tailored to extract complementary feature patterns from heterogeneous feature extraction methodologies. The reconstructed feature ensemble is then fed into an XGBoost (Chen and Guestrin 2016) classifier, which demonstrates superior predictive performance compared to traditional Random Forests due to its enhanced resistance to overfitting through randomized feature subspace partitioning. Experimental validation across six independent cell-line datasets reveals that our stacking strategy not only effectively enhances feature representation but also outperforms existing EPI prediction models with respect to both accuracy and generalizability. Notably, the ERT-based ensemble achieved a mean AUROC score of 0.909, comparable to methods such as ChINN and SPEID, while maintaining computational efficiency. These results underscore the effectiveness of our approach in synergizing multi-modal feature integration with robust ensemble learning for EPI prediction.

## 2 Methods

### 2.1 Dataset

In this work, we utilized the same enhancer–promoter interaction (EPI) dataset previously employed by TargetFinder to evaluate our model and benchmark it against other methods. The dataset includes EPIs derived from six human cell types: GM12878 (lymphoblastoid), HUVEC (umbilical vein endothelial), HeLa-S3 (cervical carcinoma), IMR90 (fetal lung fibroblasts), K562 (leukemia-derived mesodermal), and NHEK (epidermal keratinocytes). Active enhancers and promoters for each cell type were identified using annotations from ENCODE and Roadmap Epigenomics, as done in TargetFinder. High-resolution genome-wide chromatin interaction data from Hi-C experiments were used to categorize enhancer–promoter pairs as interacting (positive) or non-interacting (negative). We directly used the EPI dataset constructed in TargetFinder, in which a 1:20 ratio of positive to negative samples was predefined. In this dataset, negative samples were subsampled within distance-matched bins to ensure comparable distributions of genomic distances between enhancers and promoters in the positive and negative sets. Detailed statistics for each cell line are summarized in Table 1.

**Table 1:** Sample distribution of all cell lines in the dataset.

Cell lines	Positive samples	Negative samples
GM12878	2113	42 200
HUVEC	1524	30 400
HeLa	1740	34 800
IMR90	1254	25 000
K562	1977	39 500
NHEK	1291	25 600

### 2.2 Feature representation of sequences

To represent sequence characteristics from multiple perspectives, we applied seven feature encoding strategies designed to capture diverse aspects of nucleotide sequence information. These encodings include k-mer frequency profiles, composition of k-spaced nucleic acid pairs (CKSNAP), dinucleotide physicochemical properties (DPCPs), trinucleotide physicochemical properties (TPCPs), pseudo dinucleotide composition (PseDNC), and nucleic acid composition (NAC) (Zou *et al*. 2023). The specific definitions and construction procedures of each encoding scheme are described in detail in the following sections.

- k-mer: k-mer analysis is a foundational computational approach for encoding DNA sequence features, which involves quantifying the frequency of consecutive nucleotide subsequences of length k (Zou *et al*. 2019). In this study, we systematically employ k-mer profiles ranging from 1-mer to 4-mer to capture hierarchical sequence characteristics. Therefore, the total feature dimension is 340 dimensions. This multi-scale strategy allows simultaneous interrogation representing genomic information across spatial scales. Each DNA sequence is transformed into a k-mer–based feature vector, formulated as follows:
  (1) $$D={\left[F(k-\mathrm{mer}),F(k-\mathrm{mer}),\ldots ,F(k-\mathrm{mer}),F(k-\mathrm{mer})\right]}^{T}$$
  Where F(⋅) represents the frequency of tetranucleotide. F(k−mer) is defined as follows:
  (2) $$F(k-\mathrm{mer})=\frac{k-\mathrm{mer}}{L-k}$$
  Where L denotes the DNA sequence’s length.
- CKSNAP: It characterizes DNA sequences by calculating the frequencies of nucleotide pairs with a fixed gap of k intervening bases. In this study, the parameter *k* is set to range from 0 to 5. For each value of *k*, the corresponding composition of gapped dinucleotide pairs is computed to form the CKSNAP feature representation. The resulting feature vector is defined as follows:
  (3) $${\left(\begin{array}{c} \frac{F_{GG}}{N},\frac{F_{AA}}{N},\frac{F_{TT}}{N},\ldots ,\frac{F_{CC}}{N} \end{array}\right)}_{16}$$
  Each feature corresponds to a specific dinucleotide pattern in the sequence. Here, $F_{pq}$ denotes the occurrence frequency of the dinucleotide pq and N represents the total number of 0-spaced dinucleotides in the sequence. As a result, CKSNAP produces a 96-dimensional feature representation.
- DPCP: The DPCP representation is computed as follows:
  (4) $$\mathrm{DPCP}(a)=f(a)*\mathrm{PCP}{\left(Y_{a}\right)}_{b}$$
  Updated physicochemical properties of di- and trinucleotides were recently introduced by Zhang *et al.* These properties are adopted in this study to derive the DPCP and TPCP features. $Y_{a}$ denotes the value of the b-th DPCP descriptor. Notably, a total of 21 DPCPs were used to construct a 336-dimensional vector.
- TPCP: TPCP is calculated as follows:
  (5) $$\mathrm{TPCP}(a)=f(a)*\mathrm{PCP}{\left(Y_{a}\right)}_{b}$$
  $Y_{a}$ represents the value of b-th TPCPs. Eventually, TPCP yields an 832-dimensional feature vector derived from 13 physicochemical properties.
- PseDNC: PseDNC features capture both local and global sequence-order information of a DNA sequence and are computed as follows:
  (6) $$P={\left(p_{1},p_{2},\ldots ,p_{16},p_{16+1},\ldots ,p_{16+\lambda }\right)}^{T}$$
   
  (7) $$\begin{array}{c} p_{k}=\{\begin{array}{cc} \frac{f_{k}}{\sum_{i=1}^{16}f_{i}+w\sum_{j=1}^{\lambda }\theta_{j}.},(1\leq k\leq 16) & \\ \frac{\omega_{k-16}}{\sum_{i=1}^{16}f_{i}+w\sum_{j=1}^{\lambda }\theta_{j}},(17\leq k\leq 16+\lambda ) & \end{array} \end{array}$$
  Where $f_{k}$ denotes the normalized dinucleotide frequency of the sequence, λ indicates the maximum correlation rank considered, ω(0-1) is a weighting parameter and $\theta_{j}$ represents the j-th tier correlation factor, defined as follows:
  (8) $$\theta_{j}=\frac{1}{L-j-1}\sum_{i=1}^{L-j-1}\Theta (R_{i}R_{i+1},R_{i+j}R_{i+j+1})$$
  Where the correlation function is defined as:
  (9) $$\Theta (R_{i}R_{i+1},R_{j}R_{j+1})=\frac{1}{\mu }\sum_{u=1}^{\mu }{\left(C_{u}(R_{i}R_{i+1})-C_{u}(R_{j}R_{j+1})\right)}^{2}$$
  Where μ represents the number of physicochemical indices. In this study, six properties—rise, roll, shift, slide, tilt, and twist—were used. $C_{u}(R_{i}R_{i+1})$ is the numerical value of the u-th physicochemical index of the dinucleotide $R_{i}R_{i+1}$ at position i We set β=2, ω=2 and generated an 18-dimensional feature vector.
- NAC: NAC is a method used for feature extraction from DNA sequences. It involves analyzing the relative abundance of the four nucleotide bases: adenine (A), cytosine (C), guanine (G), and thymine (T). By calculating the composition percentages of each base within a given DNA sequence, NAC provides insights into the sequence’s characteristics, such as GC content and overall nucleotide distribution.

### 2.3 Stacking structure

**Figure 1:**
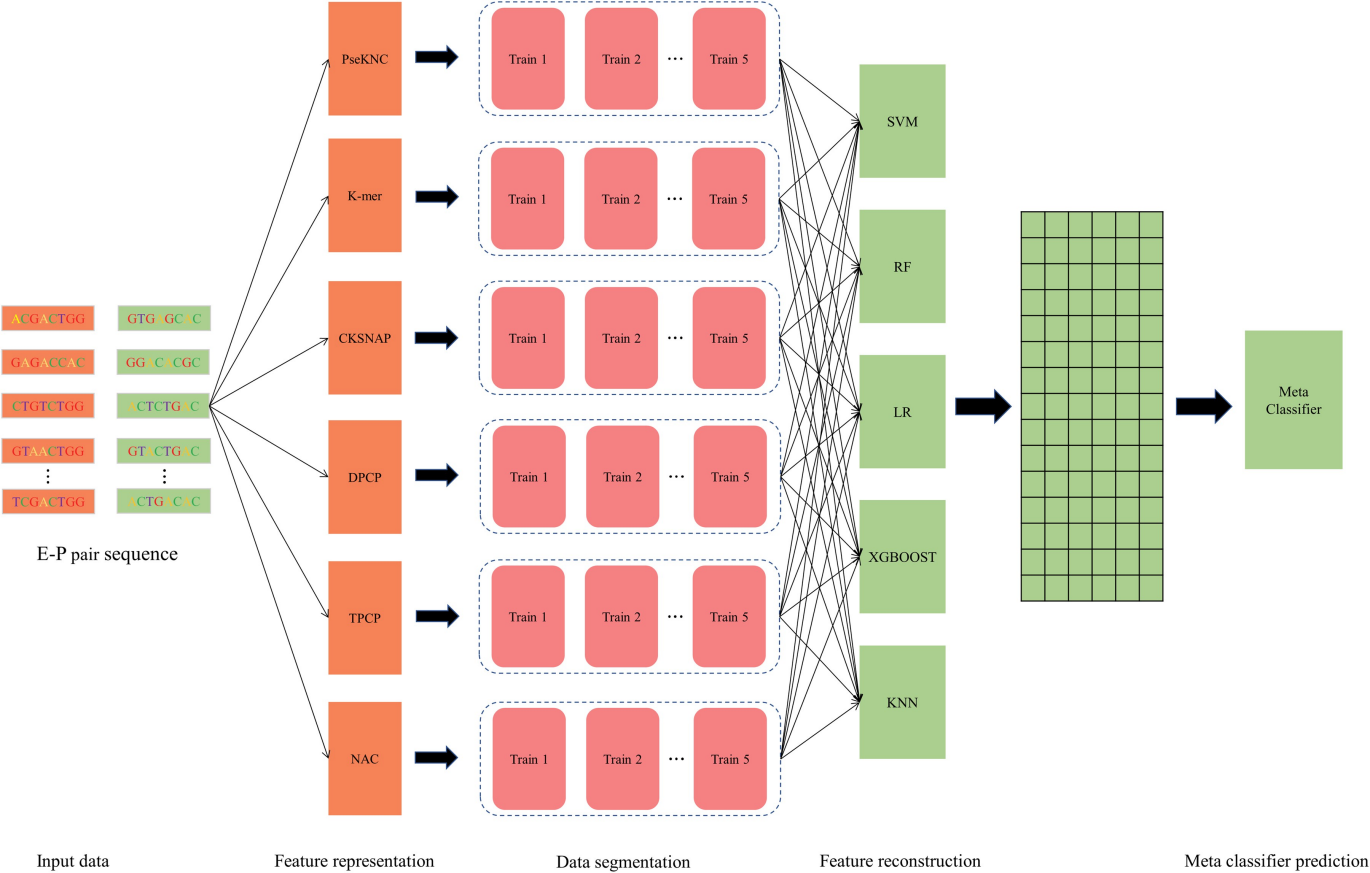
Stacked Learning Framework for Predicting EPIs.

In this work, we propose a stacking-based ensemble learning framework for cell-specific EPI prediction using only sequence-derived information. The overall architecture of the framework is illustrated in Fig. 1. While the procedures for data preprocessing and feature construction have been described previously, this section concentrates on the model design. Specifically, five strong base classifiers are combined with six types of sequence-based feature encodings, resulting in 30 first-level models. The output probabilities produced by these base models are subsequently concatenated and fed into a multilayer perceptron (MLP), which acts as a second-level meta-learner. The final predictions are obtained from the output of this meta-model.

### 2.4 Conventional classifiers

Several widely used machine learning algorithms were employed for classification in this study, including XGBoost, logistic Regression (LR) (LaValley 2008), random forest (RF), support vector machine (SVM), and k-nearest neighbors (KNN) (Kramer 2013). XGBoost is an ensemble method based on gradient boosting, LR is a linear model for probabilistic classification, RF constructs an ensemble of decision trees using bagging, SVM relies on kernel functions (Zhu *et al*. 2023) and seeks an optimal separating hyperplane in feature space, and KNN assigns labels to samples based on the similarity to their nearest neighbors. Detailed algorithmic descriptions and implementation specifics are wprovided in Supplementary Note 1, available as supplementary data at *Bioinformatics* online.

### 2.5 Model training

In this study, the dataset’s characteristic ratio of positive to negative samples is 1:20 across six cell lines. In supervised learning paradigms, models tend to prioritize the majority class, which often compromises the prediction accuracy for the minority class. To address this issue, data augmentation techniques were applied to the training set, and model performance was evaluated on an independent test set.

1. To ensure a fair comparison with existing methodologies such as EPI-VAN, we delineate the detailed training procedures of our ensemble models across different cell lines as follows:
2. Construct a training dataset D for each individual cell line, and employ data augmentation algorithms to enhance dataset D, resulting in a balanced training set $D^{\prime }$.
3. Utilize a stacking strategy to reconstruct the test dataset based on the balanced training set $D^{\prime }$.
4. Apply a stacking strategy under 5-fold cross-validation (5-CV) to further refine the balanced training dataset $D^{\prime }$.
5. Evaluate base classifiers on the newly reconstructed feature set obtained from the preceding step.
6. Perform predictions for each cell line and conduct cross-cell line validation experiments.

### 2.6 Evaluation metrics

Considering the pronounced class imbalance in the dataset employed in this study, we adopted two standard metrics to evaluate model performance: the Area Under the Receiver Operating Characteristic Curve (AUROC) ( Hanley and McNeil 1982, Wang *et al*. 2023, Chen *et al*. 2025) and the Area Under the Precision-Recall Curve (AUPR) (Davis and Goadrich 2006). The Receiver Operating Characteristic (ROC) curve illustrates how the true positive rate (sensitivity) varies with the false positive rate (1 − specificity) across different threshold settings. The AUROC quantifies the area under this curve, where values closer to 1 (corresponding to the upper-left corner of the plot) indicate stronger predictive ability. Because the ROC curve is largely independent of the proportion of positive and negative samples, AUROC is particularly suitable for evaluating models on imbalanced binary classification tasks. In contrast, the precision-recall curve captures the relationship between precision (*y*-axis) and recall (*x*-axis), focusing specifically on the performance regarding positive samples. The AUPR measures the area beneath this curve, with values approaching 1 (toward the upper-right corner) reflecting better predictive performance.

## 3 Results and discussion

### 3.1 The proposed stacking framework outperforms the state‑of‑the‑art methods

To rigorously evaluate the performance of our proposed stacking framework, we conducted a comprehensive comparison against four state-of-the-art methods: PEP-WORD, SPEID, SIMCNN, and EPIANN. All models were trained and validated using identical datasets across six distinct cell-line cohorts, ensuring methodological consistency. The training protocols for baseline models were strictly replicated according to their original methodologies. The discriminative performance metrics—AUROC and AUPR—for our stacking model and the comparator models are summarized in Tables 2 and 3, respectively.

**Table 2:** AUROC comparison of different models across cell lines.

Model	GM12878	HUVEC	HeLa	IMR90	K562	NHEK
SPEID	0.916	0.904	0.923	0.915	0.922	0.950
PEP-WORD	0.842	0.845	0.843	0.898	0.883	0.917
EPIANN	0.919	0.918	0.924	0.945	0.943	0.959
SIMCNN	0.941	0.933	0.949	0.951	0.943	0.962
ours	0.909	0.876	0.915	0.854	0.923	0.975

**Table 3:** AUPR comparison of different models across cell lines.

Model	GM12878		HUVEC	HeLa	IMR90	K562	NHEK
SPEID		0.773	0.523	0.797	0.732	0.771	0.852
PEP-WORD		0.807	0.760	0.803	0.868	0.836	0.880
EPIANN		0.723	0.616	0.702	0.770	0.673	0.861
SIMCNN		0.706	0.640	0.737	0.737	0.679	0.882
ours		0.772	0.649	0.732	0.725	0.750	0.899

In terms of AUROC, our stacking model exhibited outstanding performance across six cell lines, achieving the highest score of 0.975 in the NHEK cell line and surpassing all other models. Following closely was SIMCNN, which recorded a score of 0.962 in NHEK. Additionally, our model achieved AUROC scores of 0.876, 0.915, and 0.923 in the HUVEC, HeLa, and K562 cell lines, respectively. Although these scores were lower than those of SIMCNN (0.933, 0.949, and 0.943), they still indicate competitive performance. These results underscore the effectiveness of our model in capturing sequence patterns critical for predicting EPI, validating the architectural soundness of its stack. Regarding AUPR, our model maintained stable results across all six cell lines, particularly excelling in the NHEK cell line with a score of 0.899, significantly higher than the second-best model, PEP-WORD, which scored 0.880. In the HeLa cell line, our model achieved an AUPR score of 0.732, although it was lower than that of PEP-WORD (0.803), it still demonstrates overall reliability. Notably, PEP-WORD consistently scored lower in AUROC compared to all other models, indicating strong precision in positive class predictions but possibly at the expense of balanced classification performance.

A comparative analysis of these results reveals that our model strikes a favorable balance between AUROC and AUPR metrics. Its architecture integrates feature extraction and ensemble learning principles, making it particularly adept at capturing the complex sequence determinants underlying enhancer-promoter interactions. These findings establish the model as a powerful and versatile framework for EPIs prediction, offering advantages with regard to accuracy and precision assessment compared to existing methods.

### 3.2 Assessment of stacking strategy’s predictive performance

**Figure 2:**
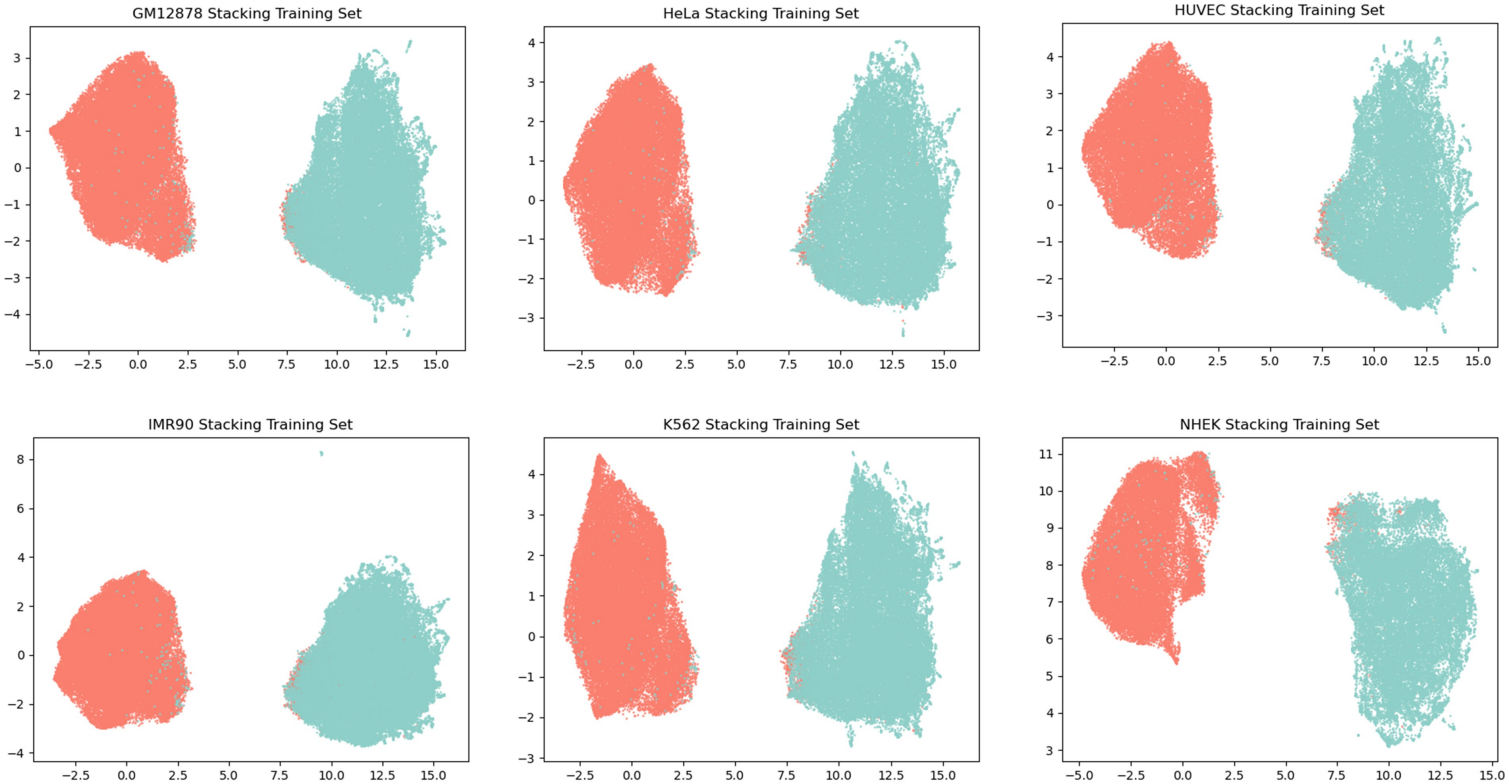
The distributions of positive and negative samples from six cell lines under the stacking framework are visualized through UMAP dimensionality reduction.

We employed a stacking ensemble strategy to reconstruct the training set features of multiple cell line datasets, including HUVEC, HeLa, K562, NHEK, GM12878, and IMR90. This multi-layered machine learning framework integrates predictions from base models through a meta-classifier, aiming to enhance feature representation and model generalization. To verify its effectiveness, we adopted a feature visualization strategy. To visualize the high-dimensional feature space, we applied the Uniform Manifold Approximation and Projection (UMAP) (McInnes *et al*. 2018) algorithm to reduce the dimensionality of the training datasets. The resulting 2D embeddings are presented in Fig. 2, which demonstrates a striking separation between positive and negative samples across all cell types. Notably, the distinct clustering patterns observed in the UMAP projections strongly suggest that our stacking ensemble approach successfully captured critical discriminative features, thereby improving the separability of complex biological datasets. This visualization not only validates the effectiveness of the stacking strategy in optimizing feature representation but also provides empirical evidence for the robustness of the model in handling heterogeneous cellular data. The clear demarcation between classes in the reduced-dimensional space indicates enhanced model interpretability and lays a solid foundation for subsequent classification tasks.

To further verify the impact of stacking on the EPIs prediction performance, we performed an ablation experiment. The stacking ensemble strategy achieved superior AUPR performance across six cell lines, outperforming all individual feature extraction methods as shown in Table 4. It is worth noting that the features reconstructed by the stack strategy are input into RF for prediction. Stacking attained the highest AUPR values in all datasets, including a remarkable 0.9138 in NHEK, surpassing the second-best method, k-mer, by 4.3%, and 0.7617 in GM12878, exceeding k-mer by 4.7%. In HUVEC, stacking maintained a 14.5% advantage over pseDNC with values of 0.7155 and 0.6243, respectively, despite lower overall AUPR values, underscoring its robustness across heterogeneous data. The average AUPR of stacking was 0.783, which was 4.1% higher than that of k-mer at 0.752, while nucleic acid composition (NAC) lagged significantly, for example, at 0.3508 in GM12878. These results highlight stacking’s ability to integrate complementary features such as k-mer, dipeptide composition, and evolutionary information, while reducing overfitting to enhance discriminative power for complex biological patterns. Among these features, k-mer representations contribute strongly due to their ability to capture local sequence context and short-range nucleotide patterns, which are often critical for distinguishing interacting from non-interacting pairs. The consistent outperformance of stacking across diverse cell lines establishes it as a robust strategy for genomic machine learning tasks, particularly in scenarios involving high-dimensional and heterogeneous data.

**Table 4:** AUPR comparison of different features across cell lines.

Model	GM12878	HUVEC	HeLa	IMR90	K562	NHEK
Kmer	0.727	0.558	0.783	0.684	0.693	0.876
CKSNAP	0.662	0.452	0.712	0.609	0.610	0.851
DPCP	0.601	0.420	0.674	0.592	0.564	0.810
TPCP	0.682	0.529	0.744	0.658	0.653	0.864
PseDNC	0.699	0.525	0.741	0.224	0.650	0.857
NAC	0.351	0.221	0.310	0.665	0.321	0.483
ours	0.762	0.626	0.793	0.716	0.735	0.899

The stacking ensemble strategy significantly enhances the prediction of Epigenetic Predictive Indicators (EPIs) by integrating complementary feature representations, achieving consistently superior performance across diverse cell lines (e.g., AUPR of 0.9138 in NHEK and 0.7617 in GM12878) compared to individual methods like k-mer or NAC. By reducing overfitting and capturing multi-scale biological patterns, stacking demonstrates robust generalization across heterogeneous datasets, establishing it as a powerful framework for precise EPIs prediction in genomic studies.

### 3.3 Analyzing the interpretability of stack strategies through SHAP

SHAP (SHapley Additive exPlanations) (Lundberg and Lee 2017) is a method grounded in game theory that provides interpretability of machine learning model outputs. It calculates a SHAP value for each feature involved in the model’s learning process, indicating the extent to which that feature affects the model’s predictions. Features with positive values contribute positively to model predictions, whereas features with negative values have a negative impact. SHAP values help generate accurate local explanations by showing how each feature affects the model’s output, the relative importance of different features, and the interactions among them.

**Figure 3:**
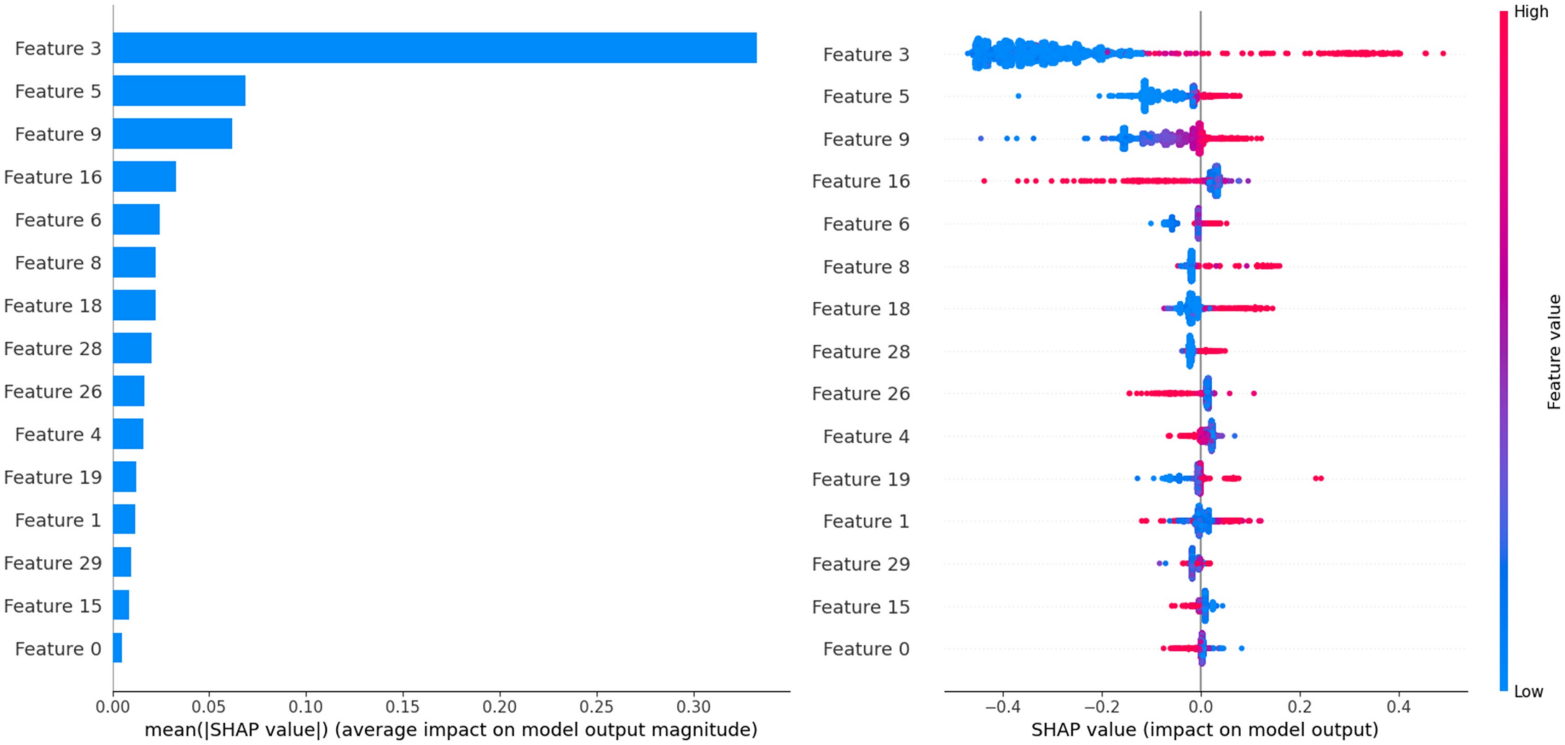
Visualization of feature importance analysis for the NHEK cell line. The left panel displays the 15 most influential features identified by SHAP value analysis, while the right panel ranks these top features by their SHAP significance scores and illustrates the directional impact of each feature’s magnitude on model predictions.

In this section, SHAP analysis was performed on the 30-dimensional DNA sequence features obtained via the stacking strategy. For each cell line, we selected and presented the top 15 features based on their SHAP value rankings, as illustrated in Fig. 3. This SHAP analysis allows us to identify which feature extraction methods yield significant features, as well as those that are less important. In the figure, the *x*-axis shows SHAP values, and the *y*-axis lists features ranked by their mean absolute SHAP values. A color gradient towards red indicates higher feature values, whereas a gradient towards blue signifies lower feature values. The width of the points represents the density of samples, reflecting areas where a large number of samples are concentrated.

Among the top-ranked features, those associated with local sequence composition patterns are observed more frequently, indicating their prominent role in model predictions. In particular, k-mer-based representations appear repeatedly among these important features, suggesting that the model effectively leverages local nucleotide composition and short-range sequence patterns for discrimination. Such features are likely to capture characteristic sequence preferences that are informative for distinguishing interacting from non-interacting pairs. In contrast, attribute-based features tend to contribute in a more complementary manner, providing additional contextual information that enhances overall prediction performance.

This analysis not only aids in understanding the model’s behavior but also enhances the interpretability of the underlying features driving the predictions, thereby contributing to the advancement of model transparency and trustworthiness in biological data analysis.

### 3.4 Validation across multiple cell lines

**Figure 4:**
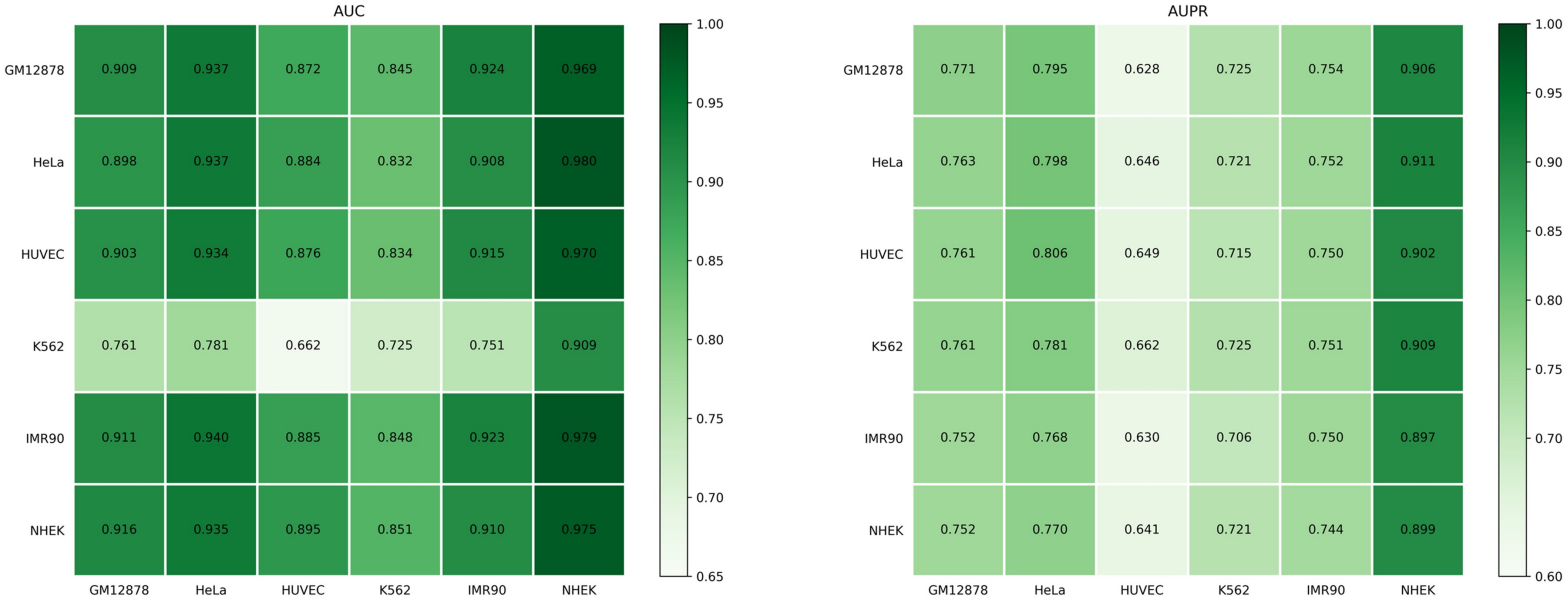
The performance of the feature set reconstructed through the stacking strategy under cross-cell-line validation.

In this selection, we conduct a comparative analysis of AUROC and AUPR across different cell lines using a stacked model, illustrated in Fig. 4. The heatmaps represent the performance metrics derived from cross-validation data for the stacked model across six cell lines. The AUROC values further reinforce this finding, with peak values of 0.980 in the HeLa cell line, illustrating the model’s strong capability to differentiate positive from negative classes. The AUPR values show that the stacked model consistently achieves high performance, with the maximum AUPR reaching 0.906 in the HUVEC cell line. This suggests that the model effectively captures the precision-recall trade-off, which is significantly important for imbalanced datasets commonly encountered in biological studies.

Overall, the heatmaps reveal that the stacked model outperforms traditional modeling approaches, as evidenced by superior AUPR and AUROC metrics across all tested cell lines. These results highlight the model’s capacity to generalize across diverse biological contexts and underscore its potential utility in predictive tasks in genomics and related fields. The consistent performance across multiple datasets validates the effectiveness of the stacking methodology, establishing it a effective tool for improving predictive accuracy in complex biological systems.

### 3.5 Comparison on UCI datasets

To assess the generalization performance of the proposed stacking framework, 5-CV was performed on six benchmark datasets from the UCI Machine Learning Repository (Asuncion and Newman 2007). The datasets do not provide predefined independent test sets, and their limited sample sizes make additional train–test splitting impractical. Consequently, all experiments were performed using 5-CV rather than separate testing procedures. The stacking-based approach was compared against several widely used classification algorithms, including RF, SVM, LR, and KNN. In addition, KHFIS performance was assessed on the same datasets. The classification accuracy (ACC) obtained from the 5-CV experiments is reported in Table 5. As a commonly adopted metric, ACC provides an intuitive measure of overall classification performance. As summarized in Table 5, the stacking learning framework achieved the best accuracy on multiple datasets, including Australian (0.8701), German Credit (0.7769), Hearts (0.8481), and Blood (0.8489). These results indicate that the proposed method maintains strong competitiveness across diverse datasets and demonstrates its applicability to classification problems beyond the original domain.

**Table 5:** ACC values of different methods on 6 UCI datasets (5-CV).

Method	SVM	KNN	RF	LR	XGBoost	ours
Australian	0.8623	0.8565	0.8638	0.8623	0.8638	0.8696
Sonar	0.8702	0.8510	0.9002	0.7502	0.9038	0.8555
German Credit	0.7630	0.7330	0.7690	0.7680	0.7660	0.7740
Blood	0.7861	0.7888	0.7687	0.7714	0.7861	0.8489
Hearts	0.8370	0.8222	0.8370	0.8407	0.8407	0.8481
Diabetes	0.7773	0.7552	0.7591	0.7734	0.7708	0.7513

## 4 Conclusions

This research introduces the stacking learning framework, a hierarchical ensemble framework designed to predict epigenetic predictive indicators (EPIs) using solely enhancer and promoter gene sequences. The method integrates six distinct feature representation techniques with five machine learning algorithms, forming a meta-classification architecture. Specifically, the stacking learning framework first constructs 30 foundational models by training combinations of feature encodings and algorithms on benchmark datasets. These models generate a 30-dimensional probability vector by aggregating their individual prediction outputs, which is then fed into a higher-level classifier to produce the final prediction.

The ensemble approach exploits the complementary strengths provided by the base models, as evidenced by superior performance metrics compared to standalone models. Notably, the stacking framework outperforms state-of-the-art methods in both computational efficiency—achieving rapid training—and predictive accuracy, particularly in AUROC scores. A key advantage lies in its flexibility: the input sequences require no strict length constraints, enabling seamless application across enhancers and promoters of diverse lengths. This adaptability positions the stacking learning framework as a promising tool for high-throughput, precise EPIs prediction tailored to specific cell lines.

## Supplementary Material

btag359_Supplementary_Data

## Data Availability

All datasets used in this study are publicly available from the original sources cited in the manuscript.
