# Supplementary material for: Predicting enhancer-promoter interactions using a stacking-based ensemble strategy
Source: Bioinformatics. 2026 Jun 4;42(6):btag359. doi: 10.1093/bioinformatics/btag359 (PMC13282077; doi:10.1093/bioinformatics/btag359)
Supplement: btag359_Supplementary_Data [file btag359_supplementary_data.docx]

- **XGBoost:** XGBoost is an efficient and scalable machine learning framework proposed by Chen and Guestrin in 2016, which is built upon the gradient boosting paradigm. A key characteristic of XGBoost is the incorporation of regularization terms into the objective function, effectively reducing overfitting during model training. To overcome the limitations of traditional greedy tree construction on large-scale datasets, XGBoost adopts an approximate splitting strategy that allows training even when the full dataset cannot reside in memory. In addition, it employs weighted quantile sketching to efficiently identify candidate split points when handling weighted samples. The framework is further optimized through sparsity-aware learning, enabling effective treatment of missing values and sparse inputs. Combined with its optimized system design—including out-of-core computation, cache-aware optimization, and support for parallel and distributed processing—XGBoost is well suited for large-scale data analysis.
- **Logistic Regression (LR):** LR is a classical statistical learning approach widely applied to binary classification problems and originates from linear regression theory. Rather than predicting continuous values, LR models the probability of class membership by passing a linear combination of input features through a logistic (sigmoid) function, which constrains outputs to the interval [0, 1]. Model parameters are typically learned by minimizing a loss function derived from maximum likelihood estimation, which penalizes incorrect predictions. Although inherently designed for binary tasks, LR can be extended to multiclass classification using techniques such as one-vs-rest or softmax-based formulations. Due to its computational efficiency, interpretability, and stable performance, LR remains a popular baseline method, particularly when the relationship between features and labels is approximately linear. Practical implementations of LR are available in common machine learning libraries, such as scikit-learn.
- **Random Forest (RF):** RF is a widely adopted ensemble learning technique that demonstrates strong performance in both classification and regression tasks. RF constructs a collection of decision trees using the bagging strategy, where each tree is trained on a bootstrapped subset of the data. Additional randomness is introduced by selecting a random subset of features at each split, which reduces correlation among trees. By aggregating predictions from multiple independent trees, RF effectively mitigates overfitting and improves robustness to noise in the data.
- **Support Vector Machine (SVM):** SVM is a traditional supervised learning algorithm grounded in the principle of structural risk minimization, originally developed in the late 1990s. SVM seeks to identify an optimal decision boundary by projecting input features into a high-dimensional Hilbert space, where a separating hyperplane with maximum margin between classes can be determined. For nonlinear classification problems, SVM relies on kernel functions[39], including linear, polynomial, and Gaussian radial basis function (RBF) kernels, to implicitly perform this transformation. In our experiments, the Gaussian RBF kernel consistently yielded the best performance and was therefore selected.
- **K-Nearest Neighbors (KNN):** KNN is an instance-based supervised learning method that classifies samples based on local similarity in the feature space. Introduced in the 1970s, KNN assigns labels to unseen samples by identifying the k closest instances according to a distance metric, such as Euclidean distance. For classification tasks, the predicted label is determined by majority voting among neighboring samples, whereas for regression tasks, predictions are obtained by averaging their values. Model behavior is highly sensitive to the choice of k, where smaller values may cause overfitting, while excessively large values can result in underfitting.
